# Potential genetic modifiers of the cystic fibrosis intestinal inflammatory phenotype on mouse chromosomes 1, 9, and 10

**DOI:** 10.1186/1471-2156-6-29

**Published:** 2005-05-27

**Authors:** Oxana Norkina, Robert C De Lisle

**Affiliations:** 1 Department of Anatomy and Cell Biology University of Kansas School of Medicine Kansas City, KS 66160 USA

## Abstract

**Background:**

Although cystic fibrosis is caused by mutations in the cystic fibrosis transmembrane conductance regulator (*CFTR*) gene, the severity of disease is highly variable indicating the influence of modifier genes. The intestines of *Cftr *deficient mice (CF mice: Cftr^*tm*1*Unc*^) are prone to obstruction by excessive mucus accumulation and are used as a model of meconium ileus and distal intestinal obstruction syndrome. This phenotype is strongly dependent on the genetic background of the mice. On the C57Bl/6 background, the majority of CF mice cannot survive on solid mouse chow, have inflammation of the small intestine, and are about 30% smaller than wild type littermates. In this work potential modifier loci of the CF intestinal phenotype were identified.

**Results:**

CF mice on a mixed genetic background (95% C57Bl/6 and 5% 129Sv) were compared to CF mice congenic on the C57Bl/6 background for several parameters of the intestinal CF phenotype. CF mice on the mixed background exhibit significantly greater survival when fed dry mouse chow, have reduced intestinal inflammation as measured by quantitative RT-PCR for marker genes, have near normal body weight gain, and have reduced mucus accumulation in the intestinal crypts. There was an indication of a gender effect for body weight gain: males did not show a significant improvement at 4 weeks of age, but were of normal weight at 8 weeks, while females showed improvement at both 4 and 8 weeks. By a preliminary genome-wide PCR allele scanning, three regions were found to be potentially associated with the milder phenotype. One on chr.1, defined by marker D1Mit36, one on chr. 9 defined by marker D9Mit90, and one on chr. 10, defined by marker D10Mit14.

**Conclusion:**

Potential modifier regions were found that have a positive impact on the inflammatory phenotype of the CF mouse small intestine and animal survival. Identification of polymorphisms in specific genes in these regions should provide important new information about genetic modifiers of the CF intestinal phenotype.

## Background

Cystic fibrosis (CF) is caused by mutations in the cystic fibrosis transmembrane conductance regulator (*CFTR*) gene [1]. Different mutations have a range of effects on the levels of CFTR protein and its proper functioning in epithelial transport of Cl^- ^and HCO_3_^- ^[2,3]. The severity of the pancreatic phenotype in human CF is well correlated with the extent of impaired CFTR function caused by specific mutations. Loss of CFTR function results in destruction of the exocrine tissue and eventual pancreatic insufficiency. On the other hand, the effects of CF on organs including the airways and intestines is less well correlated with specific *CFTR *mutations and their effects on CFTR protein function [4-8]. This indicates that other genes are likely to be important as modifiers of the CF phenotype.

With the exception of pancreatic insufficiency resulting in impaired digestion, other aspects of CF are less readily related to loss of CFTR function. Nutritional problems can persist even with adequate oral enzyme supplementation [9] and neutralization of gastric acid to improve lipase function [10], and may involve both impaired digestion and absorption of nutrients [11]. Inadequate absorption or assimilation of nutrients appears to be of greater importance because even with adequate oral enzyme supplementation nutrition is rarely fully corrected [11]. There is also excessive mucus accumulation in the CF intestine, and inappropriate inflammation is common [12]. Mucus is involved in obstruction of the gut which occurs frequently in CF infants (called meconium ileus, MI) and adults (called distal intestinal obstruction syndrome, DIOS) [11,13]. And, similar to CF airways, there is also an inflammation of the CF intestines [14,15]. These changes are less directly related to specific mutations in the *CFTR *gene and are likely related to other differences in individual genetic makeup.

Previous work using human patients and genetically altered mice has identified some modifier genes and have advanced our understanding of CF pathophysiology [4]. In one study using CF mice on different genetic backgrounds, a region on mouse chromosome 7 was shown to ameliorate intestinal blockage and the effect was in part due to a calcium-regulated Cl-channel which compensated for loss of CFTR function [16,17]. Marker haplotypes of the syntenic region of human chromosome 19q13 were also shown to be associated with the risk of MI in CF patients [18]. In other work, a region on mouse chr. 6 was strongly associated with lung inflammation, consisting of mononuclear cell interstitial infiltration and fibrosis in CF mouse airways; and other loci on chr. 1, 2, 10, and 17 were also linked to the airway phenotype [19].

In this work, CF mice on a mixed strain background were found to have a less severe CF phenotype compared to CF mice congenic on the C57Bl/6 background. There were no differences in the pancreatic phenotype comparing CF mice on the different backgrounds [20]. However, mice on the mixed background seemed more robust than CF mice on the B6 background which prompted us to characterize them in greater detail. Genome wide allele scanning was used to begin identification of regions associated with the less severe intestinal phenotype. Future identification of specific genes should further our understanding of the complex intestinal CF phenotype.

## Results and discussion

### CF mice on the mixed background have improved body weight gain

**Figure 1:**
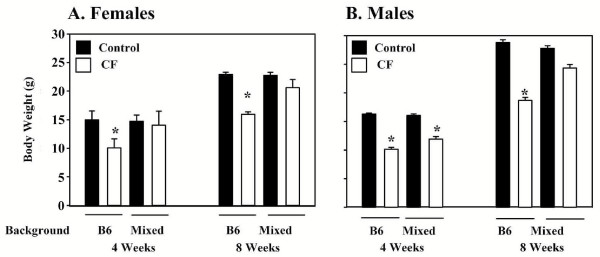
*Effect of genetic background on body weights of CF mice*. (A) Female and (B) male mice were weighed at 4 and 8 weeks of age. Data are means ± SEM. (*) *P *< 0.001 vs other three groups by ANOVA with a post-hoc Tukey's test. There were no significant differences for any of the other comparisons. Data were obtained from: 65 wild type and 26 CF B6 females, 36 wild type and 8 CF mixed background females, 68 wild type and 30 CF B6 males, and 37 wild type and 6 CF mixed background males.

Body mass was recorded for male and female mice at 4 and 8 weeks of age. On the B6 background, female and male mice are about 30% smaller than wild type mice at both 4 and 8 weeks of age (Fig. 1A, B). By comparison, female CF mice on the mixed background were not significantly smaller than wild type mice at 4 weeks of age (Fig. 1A). The improved body weight of female CF mice on the mixed background was maintained at 8 weeks of age. Male CF mice on the mixed background at 4 weeks of age were significantly smaller than wild type and not significantly larger than CF mice congenic on the B6 background (Fig. 1B). By 8 weeks of age, CF males on the mixed background were 12% smaller but this was not significantly different compared to wild type mice (Fig. 1B).

### CF mice on the mixed background have reduced expression of inflammatory markers

Previous work showed that CF mice on the B6 background have an innate-type inflammation of the small intestine [21]. To determine whether the mixed genetic background affected expression of inflammatory marker genes, quantitative, real-time RT-PCR was used to measure gene expression. Expression of the following genes was compared in wild type and CF mice on the different genetic backgrounds. Mast cell protease 2 (*Mcpt2*) is a marker of differentiated mast cells [22] and mast cells are more abundant in the B6 CF mouse intestine. Leucine-rich α2 glycoprotein (*Lrg1*, [23]) is a marker of differentiating neutrophils, which are more numerous in the B6 CF mouse intestine. The same gene is also known as leucine-rich high endothelial cell glycoprotein (*Lrhg*) and has been shown to be a marker of high endothelial venules (HEV) [24] which increase in tissues during inflammation [25,26]. Hematopoietic cell transcript 1 (*HemT1*, [27]) is a marker of blood cell proliferation and its expression is strongly elevated in the B6 CF mouse small intestine. Serum amyloid A3 (*SAA3*, [28]) is an acute phase gene and its expression in villus epithelial cells is increased in the B6 CF intestine. Suppressor of cytokine signaling 3 (*SOCS3*, [29]) is an anti-inflammatory gene that interacts with the JAK-STAT pathway and its expression in increased in the B6 CF intestine. *Muclin *(also known as *dmbt1*, [30]) expression is upregulated in the B6 CF intestine; it is a cell surface glycoprotein postulated to be an epithelial protective molecule [21,31].

**Figure 2:**
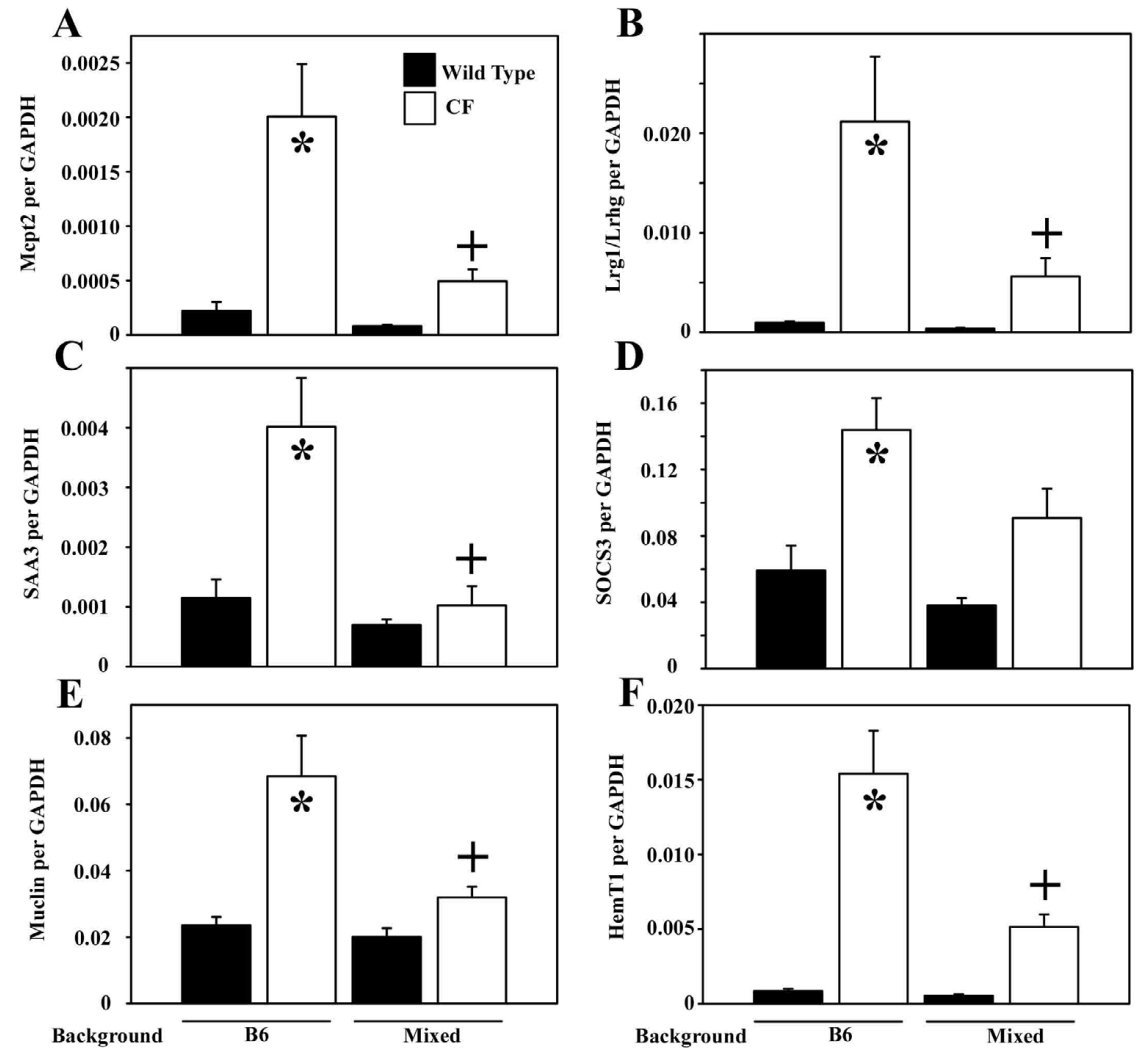
*Effect of genetic background on inflammatory gene expression in CF mouse small intestine*. RNA expression levels were determined by quantitative real-time RT-PCR using gene-specific primers. Data are expressed relative to GAPDH mRNA, which does not vary between wild type and CF mice. Data are means ± SEM. (*) CF vs wild type on the B6 background, *P *< 0.005; (+) CF on the mixed background vs CF on the B6 background, *P *< 0.05 by ANOVA with a post-hoc Tukey's test. There were no significant differences for any of the 6 genes comparing: wild type B6 mice vs wild type mixed background; or CF mice on the mixed background vs wild type on either background. There were 8–11 samples analyzed per group for each gene.

Consistent with previous results, *Mcpt2 *was increased in CF mice on the B6 background by over 9-fold compared to wild type (Fig. 2A). By contrast, there was not a significant difference in *Mcpt2 *expression between CF and wild type on the mixed background (Fig. 2A). *Lrg1/Lrhg *expression was increased more than 20-fold in CF mice on the B6 background compared to wild type, but there was no significant difference between CF and wild type on the mixed background (Fig. 2B). *SAA3 *mRNA was about 3.5-fold increased in CF mice on the B6 background, but was not significantly different compared to wild type on the mixed background (Fig. 2C). *SOCS3 *was more than 2-fold increased in CF mice on the B6 background compared to wild type, and on the mixed background was only 1.5-fold greater than wild type, and the difference was not significant (Fig. 2D). *Muclin *is overexpressed almost 3-fold in the CF intestine on the B6 background, but on the mixed background the expression level in CF mice was not significantly different than wild type (Fig. 2E). Finally, *HemT1 *was overexpressed almost 20-fold in B6 CF mice compared to wild type, and on the mixed background the CF expression level was not statistically different from wild type (Fig. 2F).

Because of the gender differences in body weight, the gene expression data were analyzed by gender but there was no significant difference between females and males. With the limited number of animals, there was also no evidence for imprinting.

### CF mice on the mixed background have less intestinal mucus accumulation

**Figure 3:**
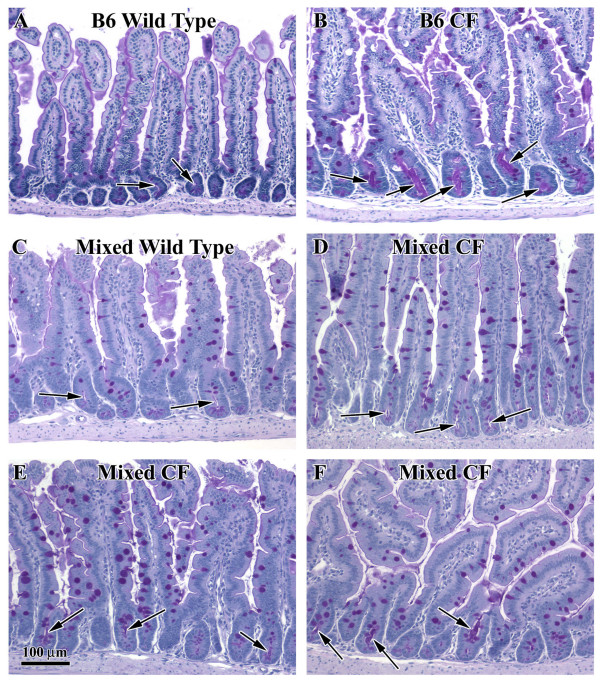
*Histological appearance of the small intestine of wild type and CF mice on the different genetic backgrounds*. Tissue was paraffin embedded and stained with PAS for neutral mucins. The sections are from the middle portion of the small intestine (A) Wild type on the B6 background. (B) CF on the B6 background. (C) Wild type on the mixed background. (D-F) CF on the mixed background. (A, C) In the wild type tissue from both backgrounds, the crypts are small and have narrow lumina (arrows). (B) In the CF tissue on the B6 background, the crypt lumina are greatly dilated and filled with PAS-reactive mucus (arrows). In some CF mice on the mixed background, the crypts are not apparently different than wild type (D), and the crypts are normal appearing (arrows). Some CF mice on the mixed background had mildly affected crypts (E), while others had greater crypt dilation and mucus accumulation (F). Overall, the CF mice on the mixed background were less severely affected compared to those on the B6 background.

The most striking histological difference in the CF mouse small intestine is the accumulation of mucus in intestinal crypts which is associated with the lethal obstruction that results in death of these mice on a standard solid chow diet [32]. Using periodic acid Schiff's staining for neutral mucins, histological analysis of small intestine tissues was performed. The wild type small intestine on both the B6 and the mixed genetic backgrounds were similar. The intestinal crypts were very small with only traces of PAS-reactivity in the lumen (Fig. 3A and 3C, respectively). The surfaces of the villus epithelium were moderately stained and goblet cells were strongly stained in the wild type tissues. In contrast, the intestine of CF mice on the B6 genetic background exhibited greatly dilated crypts filled with PAS-reactive mucus (Fig. 3B). CF mice on the mixed background had less mucus accumulation than CF mice on the B6 background (Fig. 3D-F). The amount of mucus in CF mice on the mixed background was variable from mouse to mouse. In some mice (Fig. 3D), only occasional crypts had accumulated mucus and the crypt lumina were not very dilated. In some mice there was more mucus in the crypts (Fig. 3E), while others had moderate mucus accumulation (Fig. 3F). A total of six CF mice on the mixed background were examined histologically, and three had little mucus, one had some mucus, and two had moderate amounts of mucus. Despite the variability, all the CF mice on the mixed background had less crypt dilation and less mucus accumulation compared to CF mice on the B6 background.

Thus, it appears that whatever the nature of the genetic differences between the two background strains, they affect secretion and accumulation of mucus in the CF small intestine. To determine if the difference could be accounted for by altered mucin gene expression, quantitative RT-PCR was used to measure mRNA expression of the intestinal goblet cell mucin gene, *Muc2*. In wild type intestine, B6 mice had 0.079 +/- 0.024 copies of *Muc2/GAPDH *(n = 8), and on the mixed background the level was 0.077 +/- 0.012 (n = 11). CF mice on the B6 background had 0.059 +/- 0.024 copies of *Muc2 *mRNA per *GAPDH *(n = 11), and on the mixed background the level was 0.056 +/- 0.010 (n = 11). Despite the increased mucus accumulation in CF, there is a slight decrease in *Muc2 *mRNA in the CF small intestine (*P *< 0.0001), as previously reported [33]. However, the milder CF phenotype on the mixed background, which includes less mucus accumulation, does not involve decreased mucin gene expression.

### CF mice on the mixed background have improved survival

As reported by others [34], the expected number of *Cftr *null mice on the B6 background that survived to weaning was significantly less than that expected from Mendelian genetics (Tables 1 and 2). The degree of significance was greater for male mice (Tables 1 and 2). In contrast, on the mixed background, the distribution of genotypes of female offspring was not significantly different from the expected (Tables 1). For male mice on the mixed background, the P-value was less significant but still different compared to the B6 males (Table 2). These data indicate that the mixed background is associated with increased survival of CF mice.

**Table 1:** Distribution of *Cftr *genotypes in female offspring from breeding *Cftr *heterozygotes on the B6 and mixed backgrounds.

	B6 Background		Mixed Background	
*Cftr*	Observed	Expected	Observed	Expected
+/+	207	175	57	47
+/-	371	350	88	94
-/-	122	175	42	47
*P*	0.00001 (0.04533)*		0.21724	

Statistical analysis was by Chi-square. (*) *P*-value if the sample size for the B6 mice is adjusted to be equal to that of the mixed background mice, assuming the same distribution of genotypes.

**Table 2:** Distribution of *Cftr *genotypes in male offspring from breeding *Cftr *heterozygotes on the B6 and mixed backgrounds.

	B6 Background		Mixed Background	
*Cftr*	Observed	Expected	Observed	Expected

+/+	240	201	57	52
+/-	431	403	115	104
-/-	134	201	36	52
*P*	<0.00001 (0.01612)*		0.03749	

Statistical analysis was by Chi-square. (*) *P*-value if the sample size for the B6 mice is adjusted to be equal to that of the mixed background mice, assuming the same distribution of genotypes.

**Figure 4:**
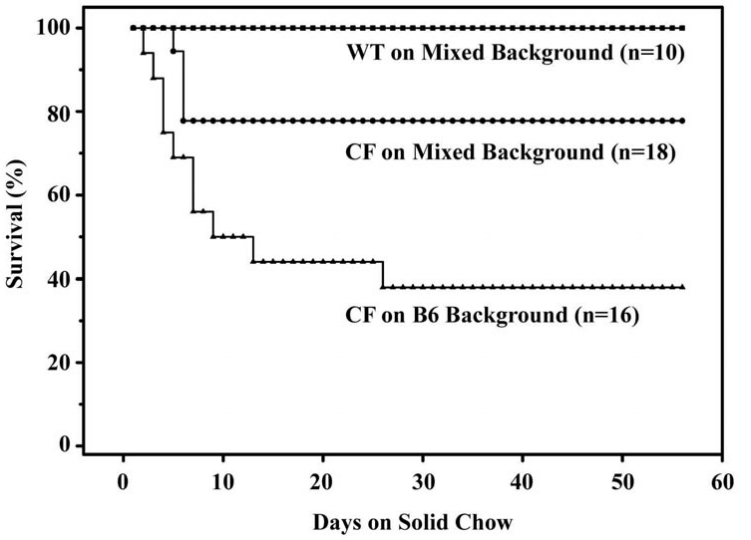
*Effect of genetic background on CF mouse survival on solid chow*. Mice were maintained on the liquid diet (Peptamen) until 8 weeks of age, at which time they were fed standard solid mouse chow for up to 8 additional weeks. Deaths were recorded as they occurred and mice in obvious distress were sacrificed and 'death' recorded as the subsequent day. By log-rank test, CF mice on the mixed background had significantly earlier death compared to wild type (*P *= 0.035), and significantly later death than CF mice on the B6 background (*P *= 0.025). CF mice on the B6 background also died significantly earlier than wild type (*P *< 0.0005).

The major cause of death in CF mice is intestinal obstruction, and intestinal obstruction is worsened when the mice are fed standard solid mouse chow [35]. Since the CF mice on the mixed background had better weight gain than on the B6 background and less mucus accumulation in the small intestine, their ability to survive on solid chow was tested. Mice were maintained on the liquid diet until 8 weeks of age, and then switched to solid chow for up to eight weeks. As shown in Fig. 4, wild type mice had 100% survival on chow, as expected. The majority of CF mice on the B6 background died within 2–3 weeks on solid chow, with about 60% mortality over the 8 week period. In contrast, only 22% of the CF mice on the mixed background died (Fig. 4).

### Identification of potential modifier loci

To determine the contributions of B6 and 129 alleles to the mixed genetic background, and to identify potential modifier regions, tail DNA was analyzed by PCR for markers of polymorphisms between these two mouse strains. Mice congenic on the B6 background, which were derived from mice originally on a B6/129 mixed background, were also analyzed to confirm they are congenic B6. Twelve CF mice on the B6 background averaged 99.5% B6 alleles. One of the twelve mice had alleles from both strains at chr.9, 40 cM. All twelve mice had both B6 and 129 markers on chromosome 6, 1 cM which is probably due to the targeted *Cftr *gene which is at chr.6, 3.1 cM [36].

Three CF mice on the mixed background were initially analyzed and were found to be 95% B6, 5% 129. A second group of 8 CF mice was analyzed and the markers used were refined to focus on the chromosomal regions showing variations from the B6 strain. The differences found from the two analyses are combined in Table 3. The differences in the mixed strain CF mice were at chr.1, 92 cM (9 of 11 mice had both B6 and 129 alleles); chr.9, 9 cM (all 11 mice had two 129 alleles); and chr.10, 65 cM (10 mice had both B6 and 129 alleles).

**Table 3:** Genome scanning analysis of CF mice on the mixed background.

Chromosome	cM	B6/B6	129/129	B6/129	Number of Mice
1	92	2	0	9	11
1	102	3	2	6	11
2	7	2	0	1	3
9	9	0	11	0	11
9	20	8	1	1	11*
9	33	5	0	3	8
9	40	1	1	1	3
10	40	6	0	2	8
10	65	0	1	10	11
11	35	5	0	3	8
11	43	6	0	5	11
12	19	6	0	2	8

The table shows the distribution of B6 and 129 alleles in mice from the two genetic backgrounds. An initial analysis was performed on 3 samples and a second analysis on 8 more samples. Only the markers that were not uniformly B6 alleles are shown. Some of the markers were refined based on the initial analysis, so not all markers were used for all 11 mice. (*) Eleven samples were analyzed but one sample was ambiguous.

Because the spacing of markers used was about 12 cM, genes within 75% of this interval on either side of the markers were looked at for potential relevance to the milder CF intestinal phenotype. None of the known chloride channels that might substitute for the missing CFTR are in the regions of the three chromosomes associated with the milder phenotype. There are several potassium channel genes in the identified regions that potentially could affect electrolyte and fluid transport: *Kcnj9 *(chr.1, 94.2 cM), *Kcnj10 *(chr.1, 93.5 cM), *Kcnj5 *(chr.9, 11 cM), and *Kcnc2 *(chr.10, 62 cM). All gene names are from the Mouse Genome Informatics website .

Inflammation is a hallmark of CF, and whether there is an inherent defect in CF that predisposes to excessive inflammation is controversial. Several genes involved in inflammation and the immune system are located in the regions of the markers identified: TNF superfamily members *Tnfsf4*, *6*, and *8 *(chr.1, 84.9–85 cM) which are involved in T cell activation [37,38]; three selectin genes (*Sele, Sell, Selp*, chr.1, 86.6 cM) which are involved in immune cell infiltration into inflamed tissues [39]; several members of immune cell surface proteins of the Slam family (*slamf1, 2, 5, 6*, and *9*; chr.1, 89.5–93.3 cM) [40]; the chemokine gene *Xcl1 *(chr.1, 87 cM) which is expressed by mast cells and recruits lymphocytes [41]; several immunoglobulin Fc receptor genes (*Fcrl3*, *Fcgr2b*, and *Fcgr3 *at chr.1, 92.3 cM; *Fcer1g *at chr.1, 93.3 cM; *Fcer1a *at chr.1, 94.2 cM); the flagellin receptor *Tlr5 *(chr.1, 98 cM); *Mmp3 *(chr.9, 1 cM) which recruits CD4+ lymphocytes [42]; *Mmp7 *(chr.9, 1 cM) which activates Paneth cell-derived cryptdins (α-defensins) [43]; *Icam1 *(chr.9, 7 cM) which is involved in lymphocyte infiltration into inflamed tissues [44]; *Kitl *(chr.10, 57 cM) which is also known as stem cell factor, and is crucial for mast cell differentiation [45]; *Im5 *(chr.10, 65 cM) which is involved in antibody-responsiveness [46]; *Lyzs *(chr.10, 66 cM) which is a Paneth cell product that digests cell walls of bacteria [47]; *Ifng *(chr.10, 67 cM) which is an important inflammatory signal in CF as well as other conditions [48]; *Il22 *(chr.10, 67 cM), a member of the anti-inflammatory IL-10 interleukin family [49]; and the *Stat2 *and *6 *genes (chr.10, 70 cM) which are important components of intracellular signaling pathways [50].

Also near the identified markers are a number of QTL associated with body weight: *Cfbw1*, CF mouse body weight at chr.1, 85 cM; *Obq9*, obesity 9 at chr.1, 88 cM; *Bw8q1*, body weight 8 at chr.1, 100 cM; *Lbm6*, lean body mass 6 at chr.9, 7.7 cM; *Bwtq4*, body weight 4 at chr.9, 8 cM; *Bgeq8*, body growth early 8 at chr.10, 57 cM; and *Pbwg5*, postnatal body weight growth 5 at chr.10, 68 cM.

Clearly, there are numerous genes in the three regions identified in this study. Because the CF mouse intestinal phenotype is characterized by an innate type immune response, with increases in mast cells and neutrophils, the genes that affect these cells are of special interest. The *Kitl *gene is crucial for differentiation of mast cells, and CF mice on the mixed background have much fewer mature mast cells than on the B6 background as revealed by less expression of *Mcpt2*. Similarly, for neutrophils the selectins and Icam1 are of interest, as these proteins are required for extravasation of neutrophils from the circulation into the inflamed tissue.

Altered immune responses may also relate to excessive mucus accumulation in the CF intestine. It is unclear why mucus accumulates to high levels in CF tissues. In part it may be due to reduced fluid secretion and a more acidic environment in the lumina of affected organs. However, there is also evidence for hypersecretion of mucus in CF [12], and it is likely that effector molecules released by mast cells and neutrophils (histamine, proteases, prostaglandins) have an important role in stimulating mucus secretion.

## Conclusion

This work demonstrated that the CF inflammatory phenotype is much less severe in mice with a small contribution of 129/Sv alleles. A preliminary analysis identified regions on chr.1, 9, and 10 are that are potentially associated with the milder phenotype. Because of the inflammation of the CF small intestine, and the possible effects of immune cells on mucus secretion, the genes in the identified regions which are involved in mast cell and neutrophil differentiation and behavior are of special interest as potential CF modifiers. Future work should focus on narrowing down these regions and determining if there are polymorphisms that affect expression of specific genes that make the CF intestinal phenotype less severe.

## Methods

### Animals

Wild type and *Cftr *null mice on two different genetic backgrounds were used in this study. One group was congenic on the C57Bl/6 (B6) background, as previously described [21]. The other group was on a mixed background of about 95% B6 and 5% 129/Sv (129). The mice on the mixed background originated as part of a recently published study [20] as follows. Mice carrying a targeted mutation of the *gastrin *gene on a mixed B6/129 background [51] were bred for eight generations onto the B6 background. The *gastrin*(+/-) mice were then crossed for six generations with *Cftr*(+/-) mice congenic on the B6 background. A genome scan at this point showed that the mice were about 95% B6 and 5% 129 (see below). These mice were bred to obtain mice wild type for both *gastrin *alleles and either *Cftr *homozygous wild type [*Cftr*(+/+)] or *Cftr *homozygous null [*Cftr*(-/-)].

Mice were genotyped at 2 weeks of age by PCR as previously described [21]. Unless otherwise stated, mice were maintained on a complete elemental liquid diet (Peptamen; Nestle Deerfield, IL) to prevent intestinal obstruction that occurs in CF mice [52]. Wild type littermates were maintained on the same diet. In some experiments, 8 week old mice were transferred onto solid mouse chow instead of Peptamen, and survival was recorded. Mice with apparent distress were sacrificed and survival on chow was recorded as the following day. Male and female mice were used at 6–16 weeks of age. Mice were kept in a specific pathogen-free facility in barrier-top cages. All procedures were approved by the University of Kansas Medical Center IACUC.

### Genetic background determination

The Genome Scanning Service of The Jackson Laboratory (Bar Harbor, ME) was used to determine the contributions of C57Bl/6 and 129/Sv strains in the interbred mice. Pieces of mouse tail were sent to The Jackson Laboratory for simple sequence length polymorphism (SSLP) PCR analysis with the DMit primers specific for B6 and 129 strain alleles . The SSLP panel consists of 108 mapped markers designed to distinguish between B6 and 129 strains. The markers are spaced 12–13 cM apart and span the nineteen autosomes.

### Measurement of gene expression

Total RNA was extracted from the entire small intestine as previously described [21]. Quantitative, real-time RT-PCR was used to measure expression of specific genes using the previously described primers [21]. Values were normalized to *GAPDH *mRNA and expression of this housekeeping gene is not altered in the CF mouse small intestine [21]. Expression of the major intestinal mucin, *Muc2*, was also measured using the forward (5'-GAC TTC GAT GGA CAC TGC TC-3') and reverse (5'-CAC GGT GTT TAT CTA CCA AC-3') primers.

### Histology

The small intestine was flushed with phosphate buffered saline and immersion fixed overnight in 4% paraformaldehyde. The tissues were then prepared for paraffin embedding and sectioning by a commercial service (HSRL, Woodstock, VA). Sections (5 μm) were stained with periodic acid Schiff's (PAS) for neutral mucins.

### Statistics

Gene expression and body weight data were compared by ANOVA with a post-hoc Tukey's analysis (Systat software, Chicago, IL). Survival data were analyzed by a log-rank test for *P *values (PEPI software, ). The distributions of genotypes of pups surviving to weaning from breeding *Cftr*(+/-) mice were compared to that expected by Mendelian genetics using Chi-square analysis. For all statistical tests, *P *< 0.05 was considered significant.

## Authors' contributions

RCD oversaw the project, performed the histological analysis, performed statistical analyses, and contributed to writing the manuscript. ON performed the quantitative RT-PCR analysis, and contributed to writing the manuscript.
